# Resistance of Hypoxic Cells to Ionizing Radiation Is Mediated in Part via Hypoxia-Induced Quiescence

**DOI:** 10.3390/cells10030610

**Published:** 2021-03-10

**Authors:** Apostolos Menegakis, Rob Klompmaker, Claire Vennin, Aina Arbusà, Maartje Damen, Bram van den Broek, Daniel Zips, Jacco van Rheenen, Lenno Krenning, René H. Medema

**Affiliations:** 1 Oncode Institute, Division of Cell Biology, The Netherlands Cancer Institute, Plesmanlaan 121, 1066 CX Amsterdam, The Netherlands; a.menegakis@nki.nl (A.M.); r.klompmaker@nki.nl (R.K.); ainaarbusa67@gmail.com (A.A.); maartje.damen@hotmail.com (M.D.); b.vd.broek@nki.nl (B.v.d.B.); l.krenning@nki.nl (L.K.); 2 Oncode Institute, Division of Molecular Pathology, The Netherlands Cancer Institute, Plesmanlaan 121, 1066 CX Amsterdam, The Netherlands; c.vennin@nki.nl (C.V.); j.v.rheenen@nki.nl (J.v.R.); 3 Bioimaging Fascility, The Netherlands Cancer Institute, Plesmanlaan 121, 1066 CX Amsterdam, The Netherlands; 4 Department of Radiation Oncology, Medical Faculty and University HospitalTübingen, Hoppe-Seyler-Str.3, 72076 Tübingen, Germany; Daniel.Zips@med.uni-tuebingen.de

**Keywords:** hypoxia, G1-arrest, quiescence, radiation resistance, HPV

## Abstract

Double strand breaks (DSBs) are highly toxic to a cell, a property that is exploited in radiation therapy. A critical component for the damage induction is cellular oxygen, making hypoxic tumor areas refractory to the efficacy of radiation treatment. During a fractionated radiation regimen, these hypoxic areas can be re-oxygenated. Nonetheless, hypoxia still constitutes a negative prognostic factor for the patient’s outcome. We hypothesized that this might be attributed to specific hypoxia-induced cellular traits that are maintained upon reoxygenation. Here, we show that reoxygenation of hypoxic non-transformed RPE-1 cells fully restored induction of DSBs but the cells remain radioresistant as a consequence of hypoxia-induced quiescence. With the use of the cell cycle indicators (FUCCI), cell cycle-specific radiation sensitivity, the cell cycle phase duration with live cell imaging, and single cell tracing were assessed. We observed that RPE-1 cells experience a longer G1 phase under hypoxia and retain a large fraction of cells that are non-cycling. Expression of HPV oncoprotein E7 prevents hypoxia-induced quiescence and abolishes the radioprotective effect. In line with this, HPV-negative cancer cell lines retain radioresistance, while HPV-positive cancer cell lines are radiosensitized upon reoxygenation. Quiescence induction in hypoxia and its HPV-driven prevention was observed in 3D multicellular spheroids. Collectively, we identify a new hypoxia-dependent radioprotective phenotype due to hypoxia-induced quiescence that accounts for a global decrease in radiosensitivity that can be retained upon reoxygenation and is absent in cells expressing oncoprotein E7.

## 1. Introduction

Solid tumors are characterized by substantial heterogeneity in oxygen availability, leading to sub-tumoral areas that are hypoxic. Tumor hypoxia is mainly caused by structurally and functionally abnormal tumor vasculature and the high oxygen consumption of the rapidly growing tumor cell population [1,2]. Tumor hypoxia has been associated with poor clinical outcome as it has been shown to confer resistance to anticancer therapies [3,4,5], cause increased genetic instability [6] and distant metastasis [7], and promote the selection and acquisition of a malignant phenotype [8,9]. Resistance of hypoxic cells to irradiation is attributed to lower induction of DNA damage in hypoxic cells. The presence of molecular oxygen at the time of irradiation generates reactive oxygen species (ROS) that can form stable non-restorable toxic adducts with the DNA molecule. In the absence of molecular oxygen, most of the DNA damage induced by free radicals can be restored chemically, thus limiting the cell-killing effect of ionizing radiation [10]. Thus, when hypoxic tumor cells are exposed to irradiation, fewer DNA double strand breaks (DSBs) are formed, as evidenced by a reduction in the numbers of γH2AX foci in hypoxic areas [11,12].

The cellular response to low oxygen levels is governed by hypoxia-induced transcription factors (HIFs) [13,14]. The HIF-transcriptional program drives the cellular adaptation to hypoxia and affects many aspects of cell biology [15]. A critical cellular response to hypoxia is the regulation of cell proliferation under hypoxic conditions by HIFs. Hypoxia inhibits the proliferation in multiple cell lines, and HIFs are both necessary and sufficient to arrest proliferation [16,17].

Cell cycle progression from the G1 to S phase is critically dependent on the activity of CDKs [18]. Their activity is required to phosphorylate and inactivate the pocket proteins (pRB, p107, and p130), causing the subsequent release of the E2F transcription factors that initiate the transcriptional program associated with S-phase entry [19]. Hypoxia, through HIF-dependent regulation of c-Myc, causes the induction of the CDK inhibitors p21 and p27, as well as expression of Cyclin D2, leading to cell cycle arrest [20,21]. In addition, HIF1a can directly interact with and decrease the activity of the minichromosome maintenance (MCM) proteins, key components for the execution of DNA replication [16,17,22]. Collectively, these data show that hypoxia, through the activity of HIFs, directly affects cell cycle progression at the G1/S phase transition.

Cellular sensitivity to irradiation displays a heterogeneous pattern across the different phases of the cell cycle [23,24]. Early studies on synchronized cell populations indicated that the most irradiation-sensitive phases are mitosis and S phase [25,26]. Recent reports accessing radiosensitivity across the cell cycle with the use of fluorescent cell cycle indicators demonstrate a G1 radioresistant phenotype [27,28]. This might have important clinical implications as G1-phase cells also exhibit resistance to chemotherapeutic agents [29,30].

Recently, it has been shown that post-hypoxic breast cancer tumor cells acquire a ROS-resistant phenotype and retain an increased expression of hypoxia-induced genes at metastatic sites despite the fast turnover of hypoxia signaling upon reoxygenation [31] Evidence for a role of persistent hypoxia-induced cell cycle arrest as a factor in therapy resistance came from another study showing that hypoxia-imprinted disseminated dormant tumor cells can reside in the lungs of mice for long periods, and evade chemotherapy [32]. Thus, inhibition of cell cycle progression, or cell cycle exit, by hypoxia might represent an additional mechanism of resistance to anticancer therapies that is independent of the amount of radiation-induced DNA damage under hypoxia, and might persist as a post-hypoxic cellular trait even upon reoxygenation. However, to date there is little know about how the hypoxia-induced cell cycle arrest might affect the resistance of hypoxic cells that reside within the primary tumor site. Here we assessed the relevance of hypoxia-induced cell cycle regulation in radiation resistance. Our data show that the radioresistance induced by hypoxia is not solely due to the oxygen effect. We demonstrate that the radioprotective phenotype is attributed to hypoxia-induced accumulation of cells in G1-arrested phase of the cell cycle, which is temporally retained after reoxygenation. Our data confirm that cell cycle position is a strong determinant of radiosensitivity, and identify a new hypoxia-dependent radioprotective phenotype in which hypoxia causes a redistribution in the cell cycle that accounts for a global decrease in radiosensitivity.

## 2. Material and Methods

### 2.1. Cell Lines and Cell Culture Conditions

hTert-immortalized retinal pigment epithelium (RPE) and derived cell lines were maintained in Advanced Dulbecco’s Modified Eagle Medium/Nutrient mixture F-12 (DMEM/F-12, Gibco, Life Technology) supplemented with 1% penicillin/streptomycin, 1% ultraglutamine and 10% fetal bovine serum (FBS, S-FBS-EU-015, Serana). RPE cells stably expressing the FUCCI sensors [33] (RPE-FUCCI) have been previously described [34,35]. RPE or RPE-FUCCI cells with doxycycline-inducible expression of E7 (RPE-E7) and RPE-FUCCI-E7 were generated by retroviral transduction of RPE (or RPE-FUCCI) cells stably expressing an ecotropic receptor and the Retro-X Tet-On Advanced Transactivator (Clontech; courtesy of Lenno Krenning) with pRetroX-tight-puro-E7 followed by puromycin selection. pBABE-E7 was a gift (Rene Bernards). pRetroX-tight-pur-E7 was obtained by PCR-mediated introduction of EcoRI and BamHI restriction sites and ligation of the product into corresponding sites of the vector. RPE-1 cells stably expressing DHB-Venus CDK2 reporter were a kind offer from the group of Tobias Meyer. Squamous cell carcinoma lines either HPV-negative (FaDu—hypopharynx, C33A—cervix)) or HPV-positive (Hela, Ca-Ski—cervix), FaDu-FUCCI cells, U2OS-FUCCI cells (osteosarcoma), and multicellular spheroids were maintained in standard DMEM supplemented with 1% penicillin/streptomycin, 1% sodium pyruvate, 2% HEPES buffer and 10% fetal bovine serum. All cell lines were routinely checked for mycoplasma. Normoxic cell culture was performed in standard humidified incubator (37 °C). An InvivO_2_300 physiological cell culture workstation (Baker) was used to maintain cells in hypoxia with 1% oxygen level. Acute hypoxic conditions (or portable for the cause of treatment) were kept in GasPak^TM^ EZ Pouch Systems (BD) that reduce oxygen levels to 0.1% within 2 h. Oxygen levels validation was done with an optical fiber and a patched oxygen sensor connected to Pyro Oxygen logger software (Pyro science) (Courtesy: Kees Jalink). All hypoxia experiments were performed with substances that have been previously de-oxygenized for at least 12 h. Serum starvation experiments in RPE cells and derivative cell lines were performed by growing cells for 72 h in confluency and subsequently cultivated in DMEM/F-12 without FBS supplementation for 48 h.

Multicellular spheroids were generated by treating a 2D cell culture with nanoshuttle solution containing magnetic nano-particles (Nanoshuttle-PL, Greiner, Bio-One GmbH) according to manufacturers guidelines. After preparation of single cell suspension, cells were seeded in non-adherent 24-well plate (Greiner, Bio-One GmbH) at a density of 100,000 cells/mL/well. To form compact structures, the plate was placed on a magnetic frame (Greiner, Bio-One GmbH) overnight. Spheroids were left to grow for three weeks and medium was changed twice per week. Expression of E7 oncoprotein was induced in RPE-E7 FUCCI spheroids by the addition of doxycylin to the culture medium. For evaluation of proliferation and hypoxic pattern, pimonidazole and BrdU were dissolved in the medium of FaDu spheroids both to a final concentration of 10 μM for 4 h. Medium was then exchanged, and spheroids were fixed in formalin for 72 h before embedding in paraffin.

### 2.2. Antibodies and Reagents

Antibodies used in the study: anti-53BP1 (dilution: 1:500, sc-629, Santa Cruz Biotechnology), anti-γH2AX (dilution: 1:1000, 05-636, Upstate Biotechnology, Millipore), secondary antibodies (dilution: 1:600, anti-mouse Alexa 568, A11004, anti-rabbit Alexa 488, A11008, Molecular Probes), anti-pimonidazole (mouse monoclonal 4.3.11.3, Natural Pharmacia International, Belmont, MA, USA, dilution 1:100), anti-BrdU (mouse monoclonal, Clone Bu20a, Dako Deutschland GmbH, Hamburg, Germany, dilution: 1:50), anti-pRB (Ser807/811, 1/1000) (Cell signaling, #9308), anti-Cyclin E1 (Cell signaling, 20808, 1/1000), anti-HIF1a (Cayman Chemicals 10006421, 1/1000), anti-alpha-tubulin (Sigma Aldrich T5168, 1/1000). Reagents used in the study: Doxycycline (D9891, Sigma-Aldrich), nocodazole (M1404, Sigma-Aldrich), Hoechst 33342 (B2261, Sigma-Aldrich), BrdU (Sigma 850187), pimonidazole (Hypoxyprobe Inc, hpi, Middlesex, Burlington, USA), SirDNA kit (SPIROCHROME), AEC kit (Signa Aldrich AEC 101), Dako Faramount aqueous mounting medium (S3025).

### 2.3. Radiation Treatment

Cells were irradiated with a Gammacell 40^®^ Exactor (Theratronics) 137Cs gamma source with a dose rate of 0.92 Gy/min.

### 2.4. Colony-Forming Assay

For the colony-forming assay (CFA), cells were grown in 10 cm dishes in either hypoxia (1%) or normoxia according to the experimental plan, in seeding densities that allow them to still be exponentially growing following 72 h of incubation. Cells were then moved to the irradiator (for the conditions OOO and HOO) or placed in GasPaks for 2 h to reach an oxygen level of 0.1% and then moved to the irradiator. Following irradiation cells were placed in a normal incubator for 24 h. Thereafter, single cell suspensions were prepared and 250 cells were seeded in 6 well plates (6 technical replicates) per dose per condition. Typically 7–10 days (according to cell line) post-seeding the plates had visible colonies. Dishes were then washed with PBS, fixed for 10 min in Methanol 100% and stained with crystal violet for at least 4 h. After drying, plates were scanned and colonies were counted manually with a manual counter application of Fiji software.

### 2.5. Western Blots

For Western Blot, cells after washing twice with PBS were lyzed in Laemmli buffer, protein was separated by SDS-PAGE and transferred to a nitrocellulose membrane (Whatman), stained with the indicated antibodies and visualized by chemiluminescence (GE Healthcare).

### 2.6. Flow Cytometry Analysis and Fluorescence-Activated Cell Sorting (FACS)

Following Bromodeoxyuridine (BrdU) (10 µM) incubation for 30 min, cells were incubated with trypsinized, washed and fixed in 70% ethanol (stored at 4 °C until further processing). After washing with PBS 0.1% Tween (PBST), DNA denaturation (2M HCl—15 min) followed by neutralization (0.1M sodium-borate buffer (pH 8.5)) and further washing with PBST the primary Rat anti-BrdU (1:250, AB6326, Abcam) (in 2% BSA TBST) was incubated for 2 h at room temperature. After washing, secondary goat anti-rat Alexa 488 (1:400, A11006, Molecular Probes) (in 2% BSA TBST) was incubated for 2 h. After washing with PBST and PBS samples were incubated with PI and RNAse at 37 °C for 20–30 min before been analyzed using BD FACSCalibur^TM^ or Attune NxT flow cytometer.

For Fluorescent-Activated Cell sorting (FACS) experiments, single cell suspension of RPE-FUCCI (or RPE-E7 FUCCI) cells was collected in phenol-red free leipovitz medium supplemented with 1% penicillin/streptomycin, 1% ultraglutamine-1, 2% FBS and 10% Hepes buffer. Cells were then sorted into 3 groups using a MoFlo Astrios Sorter^TM^ (Beckman Coulter Life Sciences) or FACS Aria™ Fusion (BD Biosciences) as indicated in Figure 2C. Cell sorting was based on the expression of the two endogenous fluorescent tags expressed by RPE FUCCI cells. The G1 sensor constituted by the monomeric Kusabira Orange fused to human Ctd1 N-terminal fragment (30-120) (mKO2-hCdt1_(30-120)_) and the S/G2 sensor constituted by the monomeric version of the Azami Green fused to human Geminin fragment (1-110) (mAG-hGem_(1-110)_) [33]. Cells with high Red and low green fluorescent were categorized as G1, low red and high green fluorescent as G2, double high fluorescent as S-phase cells, and double low fluorescent as early G1 (Figure 2C). Clonogenic capacity of RPE-FUCCI cells with similar timing post- mitosis, was assessed in cells that were either mock-treated or treated for 1 h and 4 h with nocodazole, a microtubule polymerization inhibitor that arrest cells in mitosis (and retain them colorless). Subsequently, cells were prepared for FACS as previously described and by utilizing the absence of the red fluorescent on the treated samples were selected in the mock-treated sample (Supplementary Figure S2). Cells were then sorted, seeded in 6-well plates irradiated and cultivated for seven days to produce colonies. Cultivation with Hoechst 33342 to visualize the DNA content ensured that only 2N G1 cells were sorted.

### 2.7. Immunofluorescence

RPE cells were cultivated on coverslips before being irradiated, and fixed with 4% formaldehyde for 15 min. After washing with PBS (3×) cell membranes were permeabilized with 0.10% TritonX100 (3 × 5 min) and blocking with 1% BSA for 30 min at room temperature was followed by the incubation of the primary antibodies (anti-γH2AX, anti-53BP1) for 1h in 37 °C. After washing (3× PBS), corresponding secondary antibodies were incubated for 90 min at room temperature. 4′,6-diamidino-2-phenylindole (DAPI) was applied for 10 min was followed by washing (3× PBS) and mounting with Fluorescence Mounting Medium (S3023, Dako).

### 2.8. Immunohistochemistry

Three consecutive 3 µm thickness cross-sections from the paraffin-embedded spheroids were deparaffinized with Xylol and rehydrated in graded alcohol series before microwaved with epitope retrieval buffer and subsequently stained for (a) anti-BrdU using the ARKTM Kit (animal research kit; Dako Deutschland GmbH, Hamburg, Germany), (b) anti-Pimonidazole using the VECTAstain Kit (Vectastain Elite ABC kit, PK-6102, Mouse IgG, Vector Laboratories, Inc., 30 Ingold Road, Burlingame, CA 94010 USA), (c) double stained using a peroxidase quenching step between the two stainings. As a final step, nuclei were counterstained with Haematoxylin (Figure 6A–C).

### 2.9. Imaging and Image Analysis

#### 2.9.1. Nuclear Foci

For nuclear foci analysis, images were acquired with a CoolSnap CCD cameras (Olympus), under a 60× (NA 1.45) lens IMT2 objective, Quad- polychroic mirror and Alexa Fluor filter sets (Dapi, FITC, A594) on a Deltavision (Applied Precision) system (GE Healthcare) with the use of SoftWoRx software. Maximum intensity projections of 7 optical sections of 0.50 μm spacing in the Z-direction were used. Per experimental condition, 15 individual images were acquired and a total of at least 70 cells per experiment were analyzed. Image analysis and data acquisition were performed in Fiji software with the utilization of an automatic foci counting macro. Upon nuclear segmentation based on the DAPI channel, the amount of foci per nucleus was assessed as previously described [34]. For analysis of the flow cytometry data, the FlowJo (version 10.6.0) were used.

#### 2.9.2. Cross-Sections of Multicellular Spheroids

Images of stained spheroid cross-section were acquired with a Zeiss Axiovert 135 microscope equipped with scanning stage (Motor control MCU 28) Axiocam 512 color camera under 20× (N.A. 0.75) objective (Zeiss) with the use of ZEN software. If necessary, a multiple-Tile scan was applied and stitched images were used. Image analysis and data acquisition for each spheroid was performed stepwise in Fiji software with the utilization of automated macros. Firstly, the BrdU-stained section and the Pimonidazole-stained section images were manually aligned. The rim of the section was automatically outlined. Afterwards, the hypoxia mask was generated by thresholding (using k-means clustering) the Pimonidazole signal, obtained after color deconvolution (H-AEC) of the RGB image. Then, distance-zones from the spheroid edge with 10 μm thickness were assigned over the whole section. Subsequently, with the use of two different macros, the intensity of BrdU signal and Pimonidazole signal and the fraction of positive pixels were assessed across the area of the spheroid cross-section. As a final step, both results of the BrdU and Pimonidazole intensity were embedded on the overlayed of the distant zones and hypoxic mask, allowing precise estimation of the location of each nucleus and the fraction of positive pixels for each marker across each distance zone (Supplementary Figure S4A–F).

### 2.10. Live Cell Imaging

Cells were plated in 96-well black Polystyrene Microplates (Corning, Thermo Fisher Scientific) and imaged with a LionheartTM FX Automated Microscope (BioTek^®^) under 10× objective (NA: 0.30) equipped with a Sony ICX 285 CCD camera, with pixel resolution of 1224 × 904 and a dynamic range of 62.43 (dB). The microscope is coupled with CO_2_ and nitrogen gassing allowing long-term cell population growth under either normoxic (21% O_2_) or hypoxic conditions (1% O_2_). SiR-DNA (50 μM) (Spirochrome) along with verapamil was added 30 min before starting the movie to ensure detection of mitotic figures and tracing of individual cells even across mitosis.

#### 2.10.1. Estimation of Cell Cycle Time with FUCCI (Sensors)

Images of RPE FUCCI and RPE-E7 FUCCI cells cultivated with SirDNA for 30 min prior to imaging were acquired in FITC, RFP and Cy5 channels in one focal plane with an image montage of 4 × 4 tiles per well for 120 h with time intervals of 30 min with Gen5 Microplate Reader and Imager Software Microscope (BioTek^®^). For image analysis, a macro tool was utilized in the Fiji software, which segments every nucleus in each emission channel, allowing the quantification of different cell populations per tile in every given timepoint. For the cause of individual cell tracing, cells just exiting the mitosis were followed until the next metaphase, and the residence within each individual cell cycle phase was scored with the use of the FUCCI system. Cells that either reside in a cell cycle phase for more than 24 h (or until the end of the movie) or exit the cell cycle without mitosis (turn from green to red without mitosis) were scored as non-cycling.

#### 2.10.2. Estimation of Quiescence Based on CDK2-Activity Reporter

Images of RPE-DHB-Venus cells were acquired in FITC (reporter) and Cy5 (SiR-DNA) channels in one focal plane with an image montage of 5 × 5 tiles per well for 120 h with time intervals of 60 min. For every condition and in each replicate a single stitched image was taken from the time-lapse and used for analysis. The time of each image was selected based on having exponential cell culture in both normoxic (36 h) and hypoxic (72 h) movies to account for the rapid proliferation of the normoxic cells. For analysis of CDK2 activity, a Fiji macro was developed to automatically quantify the ratio of cytoplasmic over nuclear signal, as follows. Nuclei were detected using the SiR-DNA channel by applying the StarDist convolutional neural network model for fluorescent nuclei [36]. A band around each nuclear ROI (1.5 µm width), constructed using CLIJ2 functions [37] was assigned as a cytoplasmic region. Before quantification, a rolling ball background subtraction (50 µm radius) was applied to the FITC channel. The activity of CDK2 was estimated as the ratio of cytoplasmic over nucleus median fluorescence signal intensity for every cell.

### 2.11. Live-Imaging of Multicellular Spheroids

Imaging of FaDu FUCCI and RPE-E7 FUCCI spheroids was performed on an inverted Leica TCS SP5 AOBS multiphoton microscope (Mannheim, Germany, Leica-microsystems.com) with a chameleon Ti:Sapphire pumped Optical Parametric Oscillator (Coherent Inc., Santa Clare, CA, www.coherent.com). GFP was excited with a wavelength of 980 nm and detected on a non-descanned detector (NDD) set at a wavelength of 495–550 nm. mCherry was excited with a wavelength of 1150 nm and detected on a NDD set at a wavelength of 600–645 nm. All images were acquired with a 25× (HCX IRAPO N.A. 0.95 WD 2.5 mm) water objective. Three-dimensional tile scans of spheroids were taken with Z-steps of 10 µm and a frame average of 4.

### 2.12. Analysis of 3D-Imaging of Spheroids

The population of green cells in these FUCCI spheroids was analyzed using a Fiji macro. First, rolling ball background subtraction was performed (100 µm radius) on both green and red channels, after which the green channel was normalized to the mean intensity of the (thresholded) red channel stack, in order to equalize the two signal strengths. Next, the two channels were added to combine all the cells into a single 3D stack. StarDist nuclei segmentation [36] was performed in separate 2D slices of this stack. (Accurate 3D segmentation was not possible because of the large distance between successive slices (10 µm)). The outline of the slice was determined by autothresholding (Huang) the 2D image, after sequentially applying a median filter and Gaussian blurred 2D (both 5 µm radius/sigma). Subsequently, the 2D distances from the centroids of all nuclei to the spheroid section rim were computed, and then recalculated to the shortest 3D distances to the spheroid edge, assuming a spherical shape (Supplementary Figure S4G). For every segmented nucleus, the green and red intensity *g* and *r* were measured, respectively. A histogram was then constructed of the fraction of cells that are positive for green signal, defined as the normalized green signal *g*/(*g* + *r*) being larger than 0.225, grouped in 10 µm distance bins along with the corresponding fraction of cells positive for red signal (1-green cell fraction).

### 2.13. Statistical Analysis

All statistical analyses were performed with Prism 8 (version 8.1.2, GraphPad Software Inc., 2017). Foci distributions for the same condition were tested with multiple *t*-tests. Differences in the foci distributions across different conditions were tested with ANOVA, using a Bonferroni correction. To assess differences between the cell cycle phase duration, the Kruskal–Wallis test (one-way ANOVA on ranks) was performed. In every case, a *p*-value < 0.05 was considered significant. Cell survival curves were fitted with polynomial linear quadratic model based on the equation:

Surviving fraction = exp^(−αD−βD2)^, (1)

where D is the radiation dose and α, β parameters of the linear and the quadratic term, respectively.

Differences between cell survival curves were tested with F-test. Null hypothesis implies that all the data points are fitted with one curve (simple model) while the alternative hypothesis implies that the data are better fitted with two curves (complicated model). If the *p*-value is low, the complicated model is statistically significantly better than the simpler model.

Association between the cell cycle behavior with the availability of oxygen, was tested with the Fisher’s exact test. The categorial (binomial) data of three independent experiments (0—successful cell cycle, 1—non-cycling) were pooled to generate contingency tables where the raw data for normoxia and hypoxia were placed.

## 3. Results

### 3.1. Hypoxic RPE Cells Retain a Radioresistant Phenotype upon Reoxygenation

**Figure 1:**
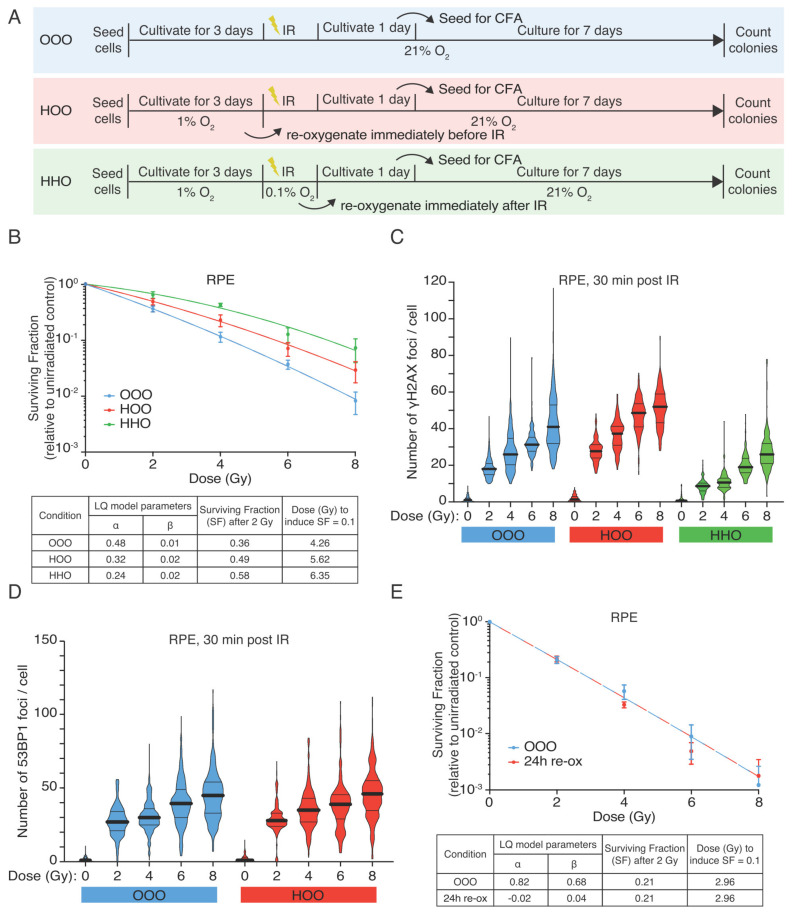
Hypoxic RPE cells retain radioresistant phenotype upon reoxygenation. (**A**) Graphical representation of the experimental design (**B**) Colony-forming assay of RPEcells. RPEcells were irradiated with graded single doses of irradiation after being constantly under normoxic conditions (blue curve—OOO), for 72 h in hypoxia (1% O_2_) and subsequently irradiated in aerated condition (time of reoxygenation in the range of 15 min) (red curve—HOO) and after 72 h hypoxia and irradiation under hypoxic conditions (0.1% O_2_—green curve HHO) before been reoxygenated immediately after IR. The data points represent the means of three independent experiments, and the error bars the 95% C.I. of the means estimation. The data were fitted with linear quadratic model, the parameters of the Linear Quadratic model (LQ) of the RPE survival curves are shown collectively for all conditions in a separate table. (**C**) DNA DSBs measured as γH2AX foci in RPEcells 30 min after irradiation with graded single doses of irradiation under normoxic (OOO, blue violin plots) and hypoxic conditions either kept in 1% O_2_ for 72 h and reoxygenated just prior to irradiation (HOO, red violin plots) or also irradiated under hypoxia (0.01% O_2_) (HHO, green violin plots). (**D**) 53BP1 foci in RPE cells 30 min post-irradiation (two of the conditions as referred in (**B**). Black solid lines represent the population mean and the dotted lines the quartiles of the data distribution (**E**) Colony-forming assay of RPE-1 cells that have been kept under hypoxic conditions (1% O_2_) for 72 h, before being reoxygenated for 24 h and then irradiated under normoxic conditions. Data represent the pool of three independent experiments, and the error bars the 95% C.I. of the means estimation. The parameters of the LQ model are depicted in a separated table.

We first assessed the effect of hypoxia on the radiation response of RPE cells, using standard colony-forming assays (CFAs) (Figure 1A). Indeed, we could observe a relative survival benefit in hypoxic cultures (Figure 1B OOO vs. HHO), a clear increase in the dose of irradiation needed to produce the same biological effect (e.g., surviving fraction of 0.1) in these different conditions (Figure 1B). Hypothesis-testing (F-test) confirmed a significant difference in survival in RPEcells irradiated in hypoxic conditions (OOO vs. HHO, F = 16.01 (*p* < 0.0001).

To examine if this difference is due to the described oxygen effect, we reoxygenated the hypoxic cultures just prior to irradiation. Surprisingly, reoxygenated hypoxic cells remained radioresistant, and no statistically significant difference was observed between the survival curve of the hypoxia-treated cells that were or were not reoxygenated at the time of irradiation (HHO and HOO, respectively) (Figure 1B). Thus, the radioprotective effects of hypoxia are not merely due to a lack of oxygen (F-test: OOO vs. HOO F = 9.07 (*p* = 0.0006), HOO vs. HHO F = 1.55 (*p* = 0.2317)) since hypoxic cells remain relatively refractory to radiation-induced cell-killing when oxygen supply is restored to normal.

Next, we tested the level of radiation-induced DSBs using immunofluorescent staining (IF) of phosphorylated histone H2AX (γH2AX), and DNA repair protein 53BP1. Irradiation in hypoxia led to a significant decrease in γH2AX over all the doses (Figure 1C). In contrast, irradiation of RPE-1 cells immediately after reoxygenation produced similar numbers of γH2AX and 53BP1 foci when comparing the normoxic and the hypoxic-reoxygenated cultures (Figure 1C,D). These results show that a brief period of reoxygenation can completely restore damage induction, consistent with the notion that the reduction in damage formation in hypoxic cells is a direct consequence of low oxygen levels. However, the radioprotective effect lingers in the reoxygenated cells (Figure 1B), indicating that the lack of oxygen cannot fully explain the radioprotective effects of hypoxia. Extending the reoxygenation duration prior to IR to 24 h abolished the radioprotective effect of hypoxia (Figure 1E), indicating that hypoxia-induced radioprotection is temporarily maintained when cells are reoxygenated.

### 3.2. The Hypoxia-Induced G1-Arrest Causes Continued Radioresistance after Reoxygenation

**Figure 2:**
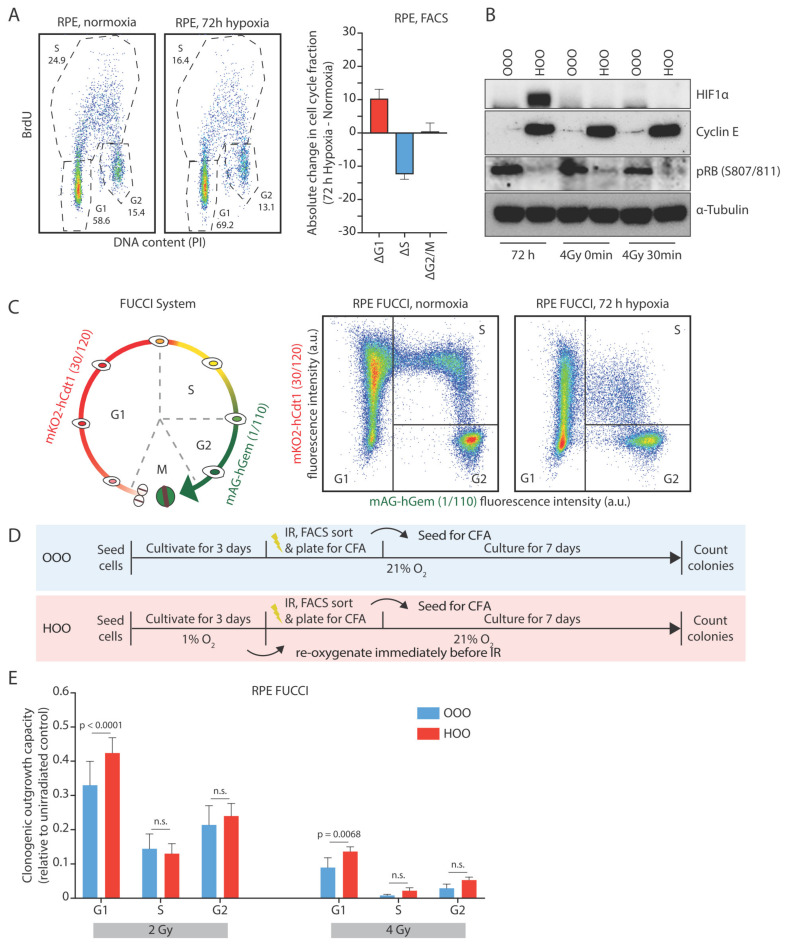
The hypoxia-induced G1-arrest causes continued radioresistance after reoxygenation. (**A**) Typical cell cycle distribution of RPE cells grown for 72 h in normoxic or hypoxic conditions (1% O_2_). The difference of each cell cycle phase fraction between hypoxic and normoxic cell cycle distribution (Δcell cycle population fraction) is depicted. (**B**) Western blot analysis of cells that have been cultured in normoxia (OO) or hypoxia (HO) before being irradiated in aerated conditions. Western blot samples were collected after culturing cells for 72 h in normoxia and hypoxia, at the time of irradiation in aerated conditions and 30 min post-IR. HIF1a as a marker of active hypoxic signaling, Cyclin E1 as a marker of quiescence, and pRB (807/811) as a marker of active transition from G1 to S phase are shown (**C**) Graphical representation of the FUCCI system. Typical cell density plots acquired from mock-irradiated RPE FUCCI cultivated either under normoxic or hypoxic conditions. The cell cycle profile based on the expression of the red and green fluorescence and the sorting of the populations is depicted (see material and methods text for more details). (**D**) Graphical representation of the experimental design. (**E**) The Surviving fraction of different cell subpopulations (indicated in **B**) after 2 and 4 Gy is shown. The bars represent the mean differences of three independent experiments, and the error bars the 95% C.I. of the means.

We hypothesized that hypoxia might exert part of its radioprotective effects via the induction of a cell cycle arrest or delay. Indeed, we observed an enrichment of hypoxic RPEcells in the G1-phase of the cell cycle and a depletion of cells in S phase in hypoxia (Figure 2A). Western blot analysis revealed that cells that have been cultured for 72 h in hypoxia have hypo-phosphorylated RB protein and high levels of Cyclin E1, which has been previously reported to increase in cells that undergo quiescence induced via serum starvation or contact inhibition [38]. Interestingly, upon short reoxygenation at the time of irradiation, the HIF1 signaling was diminished, but quiescence markers still remained high in previously hypoxic cells, further indicating that the observed radioresistance is not arising from low oxygen conditions in the cell culture (Figure 2B).

Analysis of cell cycle distribution of live RPE FUCCI cells that were previously exposed to hypoxia also showed an increased fraction of G1-phase cells compared to their normoxic counterparts (Figure 2C, Supplementary Figure S1A). Reoxygenation for twenty-four hours restored the normal cell cycle distribution (Supplementary Figure S1B). These results show that RPE cells exposed to hypoxia accumulate in G1 and slow down their proliferation rate, a phenomenon that is abrogated over time when cells are returned to normal oxygen levels.

Since cells in different cell cycle phases exhibit substantial differences in their radiation sensitivity [27,28], we hypothesized that the persistence of radiation resistance upon a short reoxygenation period might arise from an increase of the G1-phase cell population. Indeed, the surviving fraction of irradiated RPE FUCCI cells was highest when the cells exposed to irradiation were in G1-phase compared to the other cell cycle phases. This difference was observed in normoxic conditions, but also in the hypoxic RPE-FUCCI. However, the G1 cells that were exposed to hypoxia prior to irradiation exhibited the highest radiation resistance (Figure 2D,E).

### 3.3. Human Papilloma Oncoprotein E7 Prevents the Hypoxia-Induced G1-Arrest and Abolishes the Radioresistance after Reoxygenation

**Figure 3:**
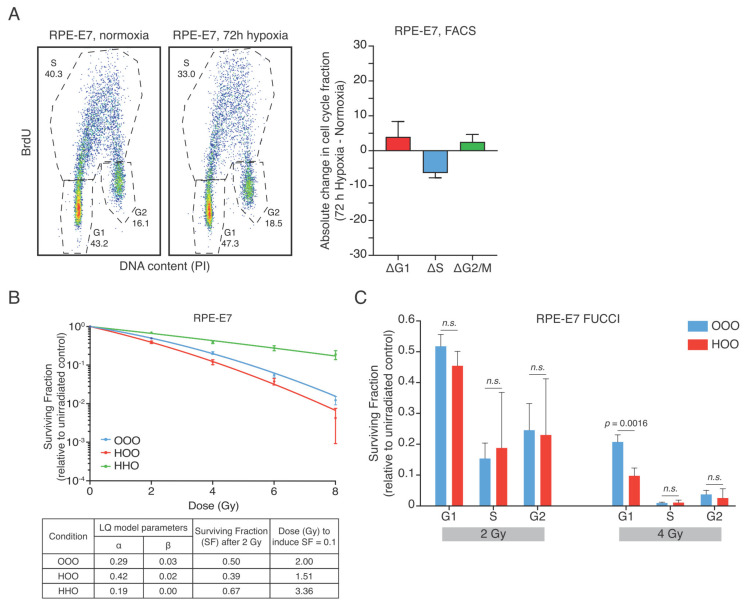
Human papilloma protein E7 prevents the hypoxia-induced G1-arrest and reverses radioresistance after reoxygenation. **(A**) Typical cell cycle distribution of RPE-E7 cells grown for 72 h in normoxic or hypoxic conditions (1% O_2_). The difference of each cell cycle phase fraction between hypoxic and normoxic cell cycle distribution (Δcell cycle population fraction) is depicted. (**B**) Colony-forming assay of RPE-E7 cells. RPE-E7 cells were irradiated with graded single doses of irradiation after being constantly under normoxic conditions (blue curve—OOO) for 72 h in hypoxia (1% O_2_) and subsequently irradiated in aerated condition (red curve—HOO) and after 72 h hypoxia and irradiation under hypoxic condition (green curve—HHO). The data were fitted with linear quadratic model, the parameters of the Linear Quadratic model (LQ) of the survival curves are shown collectively for all conditions in a separate table. (Experimental design as in Figure 1A). (**C**) Surviving fraction of different cell cycle phase subpopulations after 2 and 4 Gy is shown. The data represent the mean of three independent experiments, and the error bars the 95% C.I. of the mean. The experimental plan is similar to Figure 2D.

If the hypoxia-induced G1-arrest causes the radioresistant phenotype to persist during a short period of reoxygenation of RPE cells, we would expect that an override of the hypoxia-induced G1-arrest could suppress the radioresistance. To test this, we used an RPE cell line in which expression of the oncoprotein E7 can be induced by the addition of doxycycline (RPE-E7). High-risk HPV E7 oncoproteins destabilize the pRb-E2F complex by proteasomal degradation of the pRB protein (through Cullin 2 ubiquitin ligase complex), leading to uncontrolled S-phase entry [39]. Indeed, hypoxia-induced accumulation of cells in G1 was significantly reduced in the E7-expressing RPE cells (Figure 3A). Also, hypoxia-induced accumulation of cells in G1-phase was reduced in RPE-E7 FUCCI cells, in contrast to what we observed in the parental RPE cells (Supplemetary Figure S1C,D).

Importantly, hypoxia caused radiosensitization in RPE-E7 cells that were irradiated after a short reoxygenation time (Figure 3B), implying that an override of the hypoxia-induced G1-arrest can ablate the radioprotection seen shortly after reoxygenation. Importantly, the radioprotection due to the oxygen effect is still present in RPE-E7 cells irradiated under hypoxia (Figure 3B). F-test results indicated that the radiosensitizing effect was significant (OOO vs. HOO F=138.6 (*p* < 0.0001)). The corresponding F-test value of the RPE cells is F = 665.5 (OOO vs. HOO (*p* < 0.0001). Next, we sorted hypoxic RPE-E7 FUCCI cells that were irradiated under normoxic conditions at different stages of the cell cycle. Similar to what we observed in RPE FUCCI cells, the G1 cells displayed the highest clonogenic survival (Figure 3C), implying that cells expressing E7 could be radioprotected if they would accumulate G1. However, in contrast to parental RPE cells, hypoxia-reoxygenation failed to promote clonogenic outgrowth after irradiation of RPE-E7 cells.

### 3.4. Hypoxia-Induced Quiescence Determines Radioresistance upon Reoxygenation

**Figure 4:**
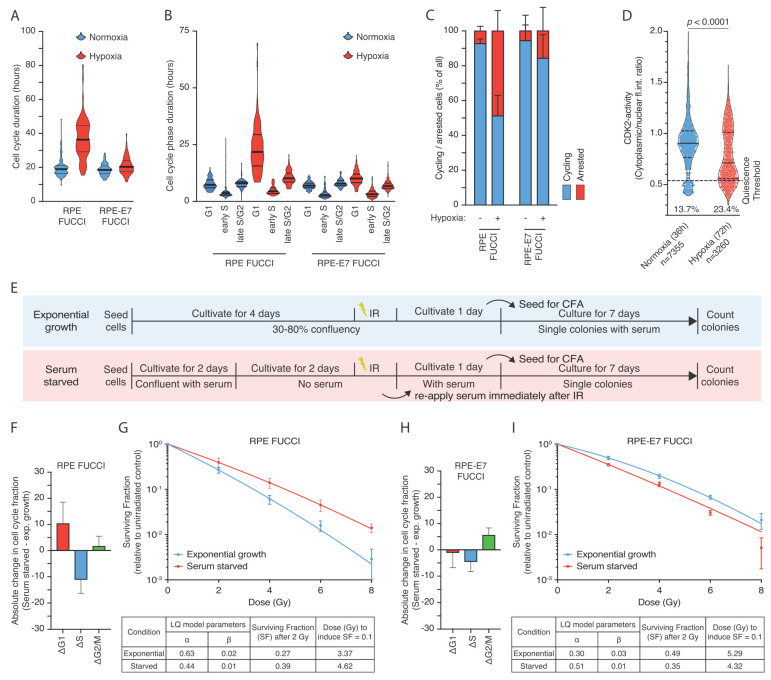
Hypoxia-induced quiescence determines radioresistance upon reoxygenation. (**A**–**B**) Total cell cycling time and the duration of RPE FUCCI and RPE-E7 FUCCI cells residing in each cell cycle phase and as analyzed based on live cell tracing throughout the cell cycle. (**C**) Analysis of cell cycle behavior in RPE FUCCI and RPE-E7 FUCCI cells in terms of cell cycle progression based on live cell tracing throughout the cell cycle. (**D**) Analysis of live cell CDK2-activity reporter. Representative distribution of cytoplasmic to nuclear intensity ratio for normoxic and hypoxic cells at similar cell densities. The horizontal line indicates the threshold levels (0.55 ratio) previously reported in cells that undergo mitogen-starvation induced quiescence [40]. The numbers indicate the total number of cells analyzed per condition and the fraction of cells with lower than 0.55 ratio. (**E**) graphical representation of the serum starvation experiments (**F**–**I**) Differences of each cell cycle phase fractions of exponentially growing and serum starved RPE FUCCI (**F**) and RPE-E7 FUCCI (**H**) cells. The difference of each cell cycle phase fraction between hypoxic and normoxic cell cycle distribution (Δcell cycle population fraction) is depicted for RPE-FUCCI (**F**) and RPE-E7 FUCCI (**H**), respectively. Colony-forming assay of RPE FUCCI (**G**) and RPE-E7 FUCCI (**I**) cells irradiated with graded single doses of irradiation after being either re-plated and growing exponentially or reaching confluency and serum starvation for 48 h at the time of irradiation.

In order to better understand the differences in cell cycle distribution induced by hypoxia, we performed live cell tracing of the cell cycle phases based on the FUCCI system in both normoxia and hypoxia. Normoxic RPE FUCCI cells exhibit a total cell cycle time of 19.94 h (95% C.I. 18.80–21.08), which was extended by hypoxia to 36.97 h (95% C.I. 34.02–39.92) (Figure 4A). Kruskal–Wallis test indicated that the differences were statistically significant (*p* < 0.0001). The respective times for RPE-E7 FUCCI cells were 18.75 h (95% C.I. 18.12–19.39) and 21.16 h (95% C.I. 20.34–21.97) in normoxia and hypoxia, respectively (Figure 4A). The main difference in the duration of cell cycle time in RPE-FUCCI cells was attributed to prolongation of G1-phase (Figure 4B). Interestingly, besides the increased duration of cells residing in G1-phase we also observed that hypoxia caused a large fraction of RPE-FUCCI cells remain arrested in a non-proliferating state (Figure 4C). Fisher’s exact test indicated that the association between this altered cell cycle behavior and the oxygen availability was highly significant (*p* < 0.0001). Importantly, E7 expression largely prevented the hypoxia-induced increase in non-cycling cells (Figure 4C). To further validate this observation, we made use of the CDK2 activity reporter in living cells, which has been previously published to be able to discriminate cell cycle progression from quiescence [40]. We observed that hypoxic cells have a significantly lower CDK2 activity reported as cytoplasmic to nuclear intensity ratio and a large fraction of cells in the range of values previously reporter to indicate quiescence induced either by serum starvation or contact inhibition [40] (Figure 4D).

Since our data show a causal link between G1-arrested cells and radioprotection, we hypothesized that any condition that would reversibly arrest cells in G1 would phenocopy the radioprotective hypoxia effect. Serum starvation has been shown to induce a transcriptional response that drive cells into quiescence [41]. Indeed, under serum starvation, most of the RPEFUCCI cells are arrested in G1-phase (Figure 4E,F, and Supplemetary Figure S2A). Quiescent RPEFUCCI cells exhibited a higher clonogenic survival compared to the exponentially growing cultures (Figure 4G). Interestingly, RPE-E7 FUCCI cells fail to display an increase in G1-arrested cells after serum starvation (Figure 4H and Supplemetary Figure S2B) and this was accompanied by prevention of the radioprotective effect (Figure 4I). These data suggest that functional impairment of the G1-arrest is enough to overcome the radioresistance induced by hypoxia or serum starvation.

To further characterize the population that retains the radioprotective effect we assessed the clonogenic capacity of cells in similar stages of G1. Normoxic and hypoxic RPE-FUCCI cells were treated with nocodazole to block cells from entering G1, while the existing G1 cells can progress through the cell cycle (Supplemetary Figure S2C,D). Using this approach, we confirmed that the early G1 cells (1–4 h after mitosis) correspond to the cells with low to intermediate expression of mKO2-hCdt1 [33]. Interestingly, when we assessed the clonogenic capacity of cycling G1 cells in normoxia and hypoxia, no difference was observed (Supplemetary Figure S2E), suggesting that the enhanced radiation resistance of the hypoxic RPE cells is due to an increase in the non-cycling G1 population.

Collectively, these data imply that the enrichment of non-cycling, dormant G1 cells, significantly contributes to the radioprotective effects of hypoxia in RPE-1 cells. Our results are in accordance with a model in which hypoxia-induced pre-conditioning of RPE- cells drives cells in a reversible state of dormancy that causes these cells to be more radiation-resistant compared to their normoxic counterparts.

### 3.5. Hypoxia-Induced G1-Arrest Determines Radioresistance upon Reoxygenation in Tumor Cell Lines Depending on HPV Status

Based on our observations in non-transformed RPE cells, we were wondering if the hypoxia-induced G1-arrest could also contribute to radiation resistance in tumor cell lines. Additionally, we reasoned that HPV-positive tumor cell lines (expressing oncoproteins E6/E7) should repond different to hypoxia than HPV-negative tumor cell lines.

**Figure 5:**
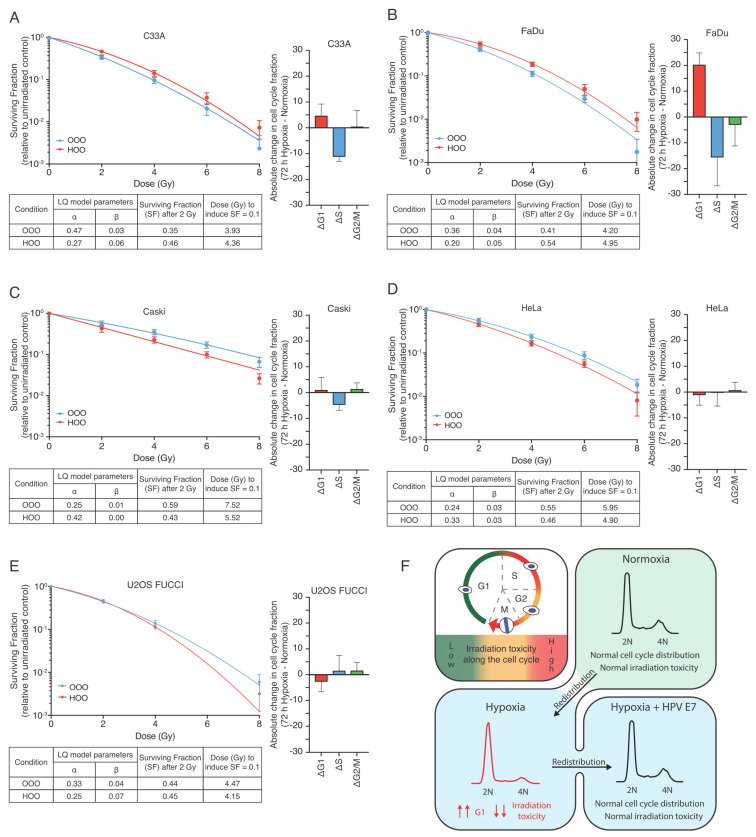
Hypoxia-induced G1-arrest determines radioresistance upon reoxygenation in tumor cell lines and it is governed by the HPV status: (**A**–**B**) Difference of each cell cycle phase fraction between hypoxic and normoxic cell cycle distribution (Δcell cycle population fraction) along with colony-forming assay of HPV-negative C33A (**A**) and FaDu (**B**). Cells were irradiated with graded single doses of irradiation after being either constantly under normoxic conditions (blue curve—OOO) or for 72 h in hypoxia (1% O_2_) and subsequently irradiated in aerated condition (red curve—HOO). (**C**–**D**) Difference of each cell cycle phase fraction between hypoxic and normoxic cell cycle distribution (Δcell cycle population fraction) along with colony-forming assay of HPV-positive Caski (**C**) and Hela (**D**) cells Similar conditions as in (**A**–**B**). (**E**) Difference of each cell cycle phase fraction between hypoxic and normoxic cell cycle distribution (Δcell cycle population fraction) along with colony-forming assay of HPV-negative U2OS cells that exhibit an aberrant G1/S transition. Cells were irradiated after being either constantly under normoxic conditions (blue curve—OOO), for 72 h in hypoxia (1% O_2_) and subsequently irradiated in aerated condition (red curve—HOO) or for 72 h in hypoxia (1% O_2_)and also irradiated under hypoxic conditions (green curve—HHO). The parameters of the Linear Quadratic model (LQ) of the survival curves are shown collectively for all conditions in separate tables. (**F**) Graphical representation of our working model depicting the main findings of the study.

Indeed, when hypoxic HPV-negative C33A and FaDu cells were reoxygenated shortly before irradiation they both exhibited a significant increase in clonogenic capacity compared to their normoxic counterparts (Figure 5A,B, Supplementary Figure S3A,B). In contrast, the clonogenic capacity was significantly reduced by hypoxia-reoxygenation in the HPV-positive cell lines Caski and Hela (Figure 5C,D, Supplementary Figure S3C,D). We hypothesized that HPV-positive cells are no longer protected directly after reoxygenation due to the fact that they fail to arrest in hypoxia. Thus, any condition that would abolish hypoxia-induced quiescence should phenocopy this observation. Indeed, using HPV-negative U2OS cells that lack functional RB [42], we observed a similar radiosensitivity upon reoxygenation compared to HPV-positive cells (Figure 5E, Supplementary Figure S3E).

Taken together, this means that hypoxia can produce a radioprotective effect that is independent of the previously established oxygen effect, but is caused by accumulation of cells in a dormant G1-phase. Importantly, this radioprotection is abolished in cells that express the E7 oncoprotein or exhibit defects in RB activation.

### 3.6. Hypoxia-Induced Cell Cycle Arrest in Multicellular Spheroids

**Figure 6:**
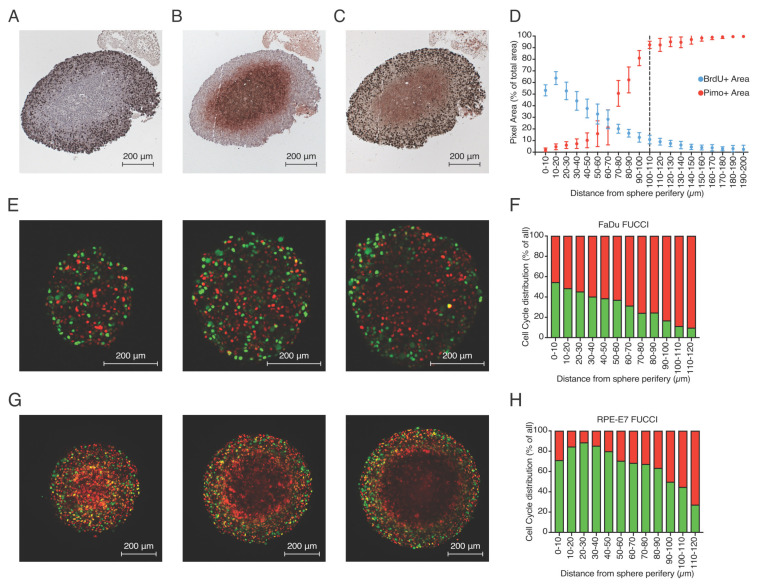
Proliferation and hypoxia profile in multicellular spheroids. (**A**–**C**) Characteristic staining of three consecutive central cross-sections (3 μm distance between them) of FaDu spheroids stained for BrdU-positive cells (**A**), pimonidazole-positive area (**B**) and double staining (**C**). (**D**) Quantification of BrdU and pimonidazole signal over different distances from the outer rim of the multiple spheroids on central cross-sections of FaDu shperoids where the anti-correlation of the two parameters is depicted. (**E**) 2-photon microsopy image of FaDu-FUCCI spheroid cross-sections at different z-levels are depicted (extracted from Supplementary Video S1). (**F**) 3-D quantification of fraction of FUCCI- expressing cells location in relation to the outer rim in FaDu-FUCCI multicellular spheroids. (**G**) 2-photon microsopy image of RPE-E7-FUCCI spheroid cross-sections at different z-levels are depicted (extracted from Supplementary Video S2). (**G**) 3-D quantification of the percentage of FUCCI-expressing cells from the outer rim in FaDu FUCCI multicellular spheroids. (**H**) 3-D quantification of the percentage of FUCCI-expressing cells from the outer rim in RPE-E7 FUCCI multicellular spheroids.

Following our observations in 2D culture, we were interested to test if our findings of hypoxia-induced quiescence would also apply to a 3D tissue culture model. Unfortunately, RPE cells failed to grow as 3D spheroids, so we were unable to directly compare this to the results obtained in monolayers of RPE cells. Therefore, we developed multicellular spheroids from FaDu tumor cells and analyzed their proliferation and hypoxia profiles across the multicellular spheroids. Three-week-old FaDu spheroids acquire a size larger than 500 μm in diameter (central cross-section), and based on the limited oxygen diffusion, develop a hypoxic core that can be visualized in central cross-section as pimonidazole-positive areas (Figure 6B, Supplementary Figure S4B). Interestingly, the addition of BrdU (as an active S-phase marker) to visualize active proliferation revealed that this is almost exclusively limited in the outer rim of the spheroid marking the pimonidazole-negative area (Figure 6A,C, Supplementary Figure S4A,C). Analysis of 10 different spheroids (Supplementary Figure S4D–F, see Section 2) revealed the anti-correlation of proliferation and hypoxia parameters in FaDu spheroids and indicated that as an average, hypoxia develops at a distance of 100–110 μm from the periphery of the spheroid (Figure 6D). Proliferation appears to be largely limited to the outer 100 μm generating a characteristic profile. Importantly, this distance is in accordance with previously reported oxygen diffusion distances from the edge of blood vessels in xenografted FaDu tumors [11].

To test if E7-expressing cells would also alter the hypoxia-induced quiescence profile in spheroids, we generated multicellular spheroids of RPE-E7 FUCCI cells and FaDu-FUCCI cells and visualized them live with 2-photon microscopy to analyze the proliferation pattern along the depth of the spheroid. As expected, based on the cross-section analysis of the FaDu spheroids, we observed that the vast majority of the inner core of FaDu-FUCCI spheroids remain in G0/G1-phase while proliferation is limited to the outer rim with increasing depth (Figure 6E, Supplementary Video S1). In sharp contrast, RPE-E7 FUCCI spheroids exhibit a more homogeneous pattern of proliferation across the whole mass of the spheroid (Figure 6G, Supplementary Video S2). Three dimentional quantification taking into account the actual distance of each nuclei from the sphere periphery (Supplementary Figure S4G) of all FUCCI- red cells and FUCCI-green cells revealed that in FaDu FUCCI spheroids the distribution of G0/G1 cells is more “skewed” towards the core of the spheroids (larger distances from spheroid edge) indicative of a gradient of diminishing proliferation (Figure 6F). In contrast, in RPE-E7 FUCCI spheroids, a more homogeneous distribution of S/G2 cells was observed for larger distances, which only declines in areas where nuclei detection was compromised due to the high compaction of the RPE-E7 FUCCI spheroids (Figure 6H). The high compaction of RPE-E7 FUCCI spheroids led to lack of both green and red nuclei detection, especially pronounced in inner core of deeper slices, and led to a background detection of red light due to higher penetration of red emission light (higher wavelength).

## 4. Discussion

Here, we show that the radioprotective effects of hypoxia are not solely due to this “oxygen effect” [10,43,44]. While we find that a brief period of reoxygenation is sufficient to fully restore the induction of DSBs to the level of normoxic RPE cells, this is insufficient to restore normal radiosensitivity in these cells.

We hypothesized that this radioresistant phenotype was a consequence of accumulation of hypoxic RPE cells in the G1-phase based on the regulatory role of HIF signaling on the G1/S transition of the cell cycle [16,17]. Indeed, analysis of the cell cycle profile revealed enrichment of hypoxic RPE cells in the G1-phase, consistent with previous reports for normal and tumor cell lines [45,46]. In line with this, we were able to show that RPE cells are most resistant to irradiation when they are in the G1-phase of the cell cycle. This contradicts earlier work using drug-synchronized cells that identified late S-phase as the most radioresistant cell cycle phase [25,26], but is consistent with more recent studies that use the FUCCI system to demonstrate that the G1 cells of a murine breast cancer model [27] or from murine melanoma cells [28] exhibited the highest radioresistance. Importantly, our study is the first to show that hypoxic G1-arrested cells exhibit even higher clonogenic capacity compared to normoxic G1 cells, even upon reoxygenation.

Interestingly, prolonged reoxygenation will fully revert this radioresistant phenotype, and this coincides with the reestablishment of a normal cell cycle distribution in RPE cells. These results are in line with previously published data for head and neck tumor cell lines, in which authors observed that 8 h of hypoxia (1%) followed by reoxygenation for 24 h prior to 8 Gy irradiation fully restores intrinsic radiosensitivity [47]. Our data are consistent with the notion that the hypoxia-induced cell cycle arrest in G1 drives radioresistance. Indeed, expression of the viral oncoprotein E7, which overrides the hypoxia-induced G1-arrest prevented the persistence of hypoxia-induced radioprotection. Furthermore, we observed that hypoxic G1 RPE-E7 cells exhibit lower clonogenic capacity compared to their normoxic counterparts, clearly different from the the radioresistance we observe in hypoxic G1-arrested RPE parental cells. Recently, it has been reported that HPV-positive cell lines enter a reversible mode of dormancy under hypoxia via downregulation of E6/E7 oncoproteins [48]. However, the authors reported that the hypoxia-induced repression of E6/E7 did not result in increased expression of p53 or Rb protein, respectively, while siRNA-mediated depletion of E6/E7 (in normoxic condition) does result in increased p53 or Rb expression. These findings suggest that repression of viral antigens in hypoxia might not be enough to lead to reactivation of E6/E7 targeted proteins, such as Rb. Our data support this observation, since we observed similar cell cycle profiles in both normoxic and hypoxic RPE-E7 cells, which is indicative of impaired Rb-activity.

Hypoxic cells have been previously reported to downregulate important proteins of the homologous recombination (HR) pathway [49,50,51], a phenomenon that has been shown to persist for up to 48 h post-reoxygenation after severe hypoxic conditions [49]. Downregulation of the HR proteins by hypoxia might render cells that remain in S and G2 phase of the cell cycle more vulnerable to irradiation upon reoxygenation. However, our experiments show that normoxic S and G2-phase cells do not have better clonogenic capacity compared to their hypoxic counterparts, indicating that repair and recovery of hypoxic S and G2-phase cells can occur normally. This could either mean that loss of HR-activity is compensated by repair via other pathways, or could be due to the downregulation of HR protein requiring severe hypoxia, higher than achieved in our set up.

Hypoxia within the tumor microenvironment can alter radiation sensitivity in diverse ways. Hypoxic cells, stabilize HIF signaling, which, in turn, alters cellular redox and promotes the usage of alternative metabolic pathways [52]. Utilization of the glycolytic pathway has been shown to lead to increased radiation resistance in different cell lines [53,54,55]. Additionally, the switch to the glycolytic pathway will increase the release of lactic acid in the microenvironment of the hypoxic cells favoring the production of L-2-hydroxyglutarate [56,57] (a-Ketoglutarate antagonist), which inhibits the prolyl-hydroxylases and can potentially stabilize HIF signaling even in the absence of low oxygen in the neighboring cells. That in turn might favor the utilization of glycolytic and or glutamine pathways which can also lead to increased radiation resistance in the surrounding cells [58,59]. Therefore, targeting the altered metabolic pathways in cancer has been proposed to improve the therapeutic outcome of radiation therapy [60,61]. On the other hand, hypoxic signaling might trigger angiogenesis [62,63], a phenomenon that will counteract hypoxia and cause reoxygenation of tumor tissue, thus enhancing radiation sensitivity. The interplay of these mechanisms might define the balance between optimal and adverse therapeutic outcome. In our study, the time and the level of hypoxia exposure remain constant throughout the experimental design. Therefore, no conclusion could be drawn on the relationship between duration and severity of hypoxia with the duration of the cell cycle arrested phenotype. However, it has been shown that prolonged cultivation of cells in hypoxia can enhance their ability to survive hypoxia and retain an arrested phenotype for long periods (beyond 24 h) after reoxygenation [64]. Additionally, the molecular mechanism by which the G1-arrest/quiescence contributes to radiation resistance is not yet understood and provides the basis for future studies. Nevertheless, our findings of persisting radioprotection of hypoxic cells upon reoxygenation, managed to un-masked an underliying radioprotective phenotype that has been commonly overlooked. Though the impact of induced DNA damage amount in hypoxic cells on cellular survival is critical, our study suggests that cell cycle position might be an important parameter that determines the fate of post-hypoxic cells.

Our observations might have important implications for radiation therapy of solid tumors in which chronic hypoxic areas commonly develop close to necrotic zones due to insufficient oxygen supply [31]. Cells within these areas need to adapt to this harsh microenvironment to survive. This involves reprogramming key cellular functions, e.g., re-wiring their intracellular metabolism, altered mitochondria function, reduced nutrient usage, and utilizing lipid and carbon metabolism [52]. Reoxygenation of those areas is a phenomenon that has been shown to occur during the course of a fractionated radiation therapy schedule both in preclinical animal models and in cancer patients [65,66]. While appreciating that tumor cells within the tumor mass can experience a wide range of oxygen fluctuation levels and cycles of hypoxia-reoxygenation that could yield a very different biological response, our data imply that cells residing in hypoxic areas within the tumors might enter a reversible quiescent state that confers an extra mechanism of radioresistance. In our setup, we focused on the radiation response of hypoxic cells and, therefore, we cannot make any conclusion regarding the interaction that hypoxic tumor cells have with stromal cells that reside within the tumor mass and has been shown to influence the therapeutic response of xenografted tumors in vivo [67] nor to the increase metastatic potential that hypoxic cells have shown to possess that can impact the therapeutic outcome [31]. Our results are in line with recent reports indicating that chronic hypoxic cells enter a dormant state [32] and further suggest that reoxygenation per se is not enough to sensitize cells that have been previously exposed to hypoxia. It is rather the redistribution of post-hypoxic, reoxygenated cells into the cell cycle that brings the cells back to their intrinsic radiation sensitivity. Persisting dormancy of hypoxic cells upon reoxygenation could render them radioresistant in consequetive radiation fractions. This phenomenon will lead to compromised radiation therapy efficiency of solid tumors and warants further investigation regarding the time-course of hypoxia-induced quiescence removal upon reoxygenation in tumors. Reoxygenation of cells in the tumor mass can be induced by irradiation as a result of radiation-induced cell death and cell cycle arrest, both of which will lower oxygen consumption. Highly oxygenated cells will suffer relatively more damage due to the oxygen effect, and, therefore, the lowered oxygen consumption will be most prominent in the cells that lie close to a blood vessel. As a result of this, the oxygen diffusion distances will increase [68]. This implies that fractionation schedules should be optimized in such a way oxygenated cells are killed effectively with a high dose per fraction (hypofractionation), while the inter-fraction interval is optimized to allow for optimal re-entry of the previously hypoxic cells into the cell cycle. Obviously, higher dose per fraction might lead to increased normal tissue toxicity, and one will need to take into account the specific tumor type in the context of critical normal tissues residing within the radiation field. Therefore, approaches that will aim to alter the hypoxic microenvironement via normalizing tumor vasculature [69] or increasing oxygen delivery to the hypoxic tumor cells by other means [70,71,72,73] might be critical in altering the adverse therapeutic outcome of hypoxic tumors with better patient selection and utilization of hypoxia-specific biomarkers. Prominent recent approaches to tackle the problem presented by hypoxic regions in a tumor aim to target the hypoxic cells with hypoxia-activated prodrugs in order to remove them prior, or during radiation therapy [74,75,76].

Our data nicely show that this transient radioprotective effect of hypoxia/reoxygenation is absent in cells expressing HPV E7. This implies that E7-positive tumors should respond better to radiation therapy, as hypoxia is expected to be less radioprotective in such tumors. This hypothesis is supported by several recent meta-analysis reports from different tumor sites that indicate better survival outcome for patients with HPV-related tumors [77,78,79]. Interestingly, while there are no differences in hypoxia extent and distribution between HPV-positive and HPV-negative head and neck tumors, evaluated with F-Miso and 15-gene hypoxia classifier, hypoxic treatment modification with nimorazole have been shown to be less effective and proved to be an unnecessary treatment option for HPV-positive tumor patients [80]. Though E7 oncoprotein expression in HPV-positive tumor cells was used in this study as a tool to bypass the G1-arrest, our data on HPV-negative tumor cells with an RB defect indicate that in genetically unstable cancer cells any pathway that can potentially lead to a premature G1/S transition and abolish the hypoxia-induced G0/G1-arrest will lead to a similar radiosensitivity phenotype upon reoxygenation, thus not limiting our findings to HPV-positive tumors.

## 5. Conclusions

Tumor hypoxia imposes the primary barrier to the efficacy of radiation therapy. This phenomenon has been mainly attributed to the lower induction of DNA double strand breaks by irradiation due to low cellular oxygen tension. Here, we identify hypoxia-induced quiescence as an additional mechanism of radiation resistance, which can be retained at least temporarily upon reoxygenation. We show that hypoxic non-transformed and cancer cells arrest in the G1 phase of the cell cycle. Brief reoxygenation fully restores the radiation-induced DNA damage to fully oxygenated levels, but surprisingly, this is not sufficient to fully restore radiosensitivity. This is due to maintenance of the G1-arrested state, a mode of radioprotection that is lost in tumor cells expressing the HPV E7 oncoprotein.

## Data Availability

Research data are stored in an institutional repository and will be shared upon request to the corresponding author. Software used in the study can be found at: https://zenodo.org/record/4591915.

## Supplementary Materials

The following are available online at https://www.mdpi.com/2073-4409/10/3/610/s1, Figure S1: Cell Cycle Distribution of RPE FUCCI and RPE-E7 FUCCI upon reoxygenation, Figure S2: Radiation Sensitivity of proliferating Normoxic and Hypoxic early-G1 cells, Figure S3: Hypoxia-induced G1 accumulation in tumor cell lines with abberant G1/S transition, Figure S4: Development of automated macro to assess proliferation and hypoxia pattern in multicellular spheroids, Video S1: FaDu_spheroid_2-Photon microscopy.
