# Supplementary material for: Resistance of Hypoxic Cells to Ionizing Radiation Is Mediated in Part via Hypoxia-Induced Quiescence
Source: Cells. 2021 Mar 10;10(3):610. doi: 10.3390/cells10030610 (PMC7998378; doi:10.3390/cells10030610)

Supplementary Figure 1 - Cell Cycle Distribution of RPE FUCCI and RPE-E7 FUCCI upon reoxygenation

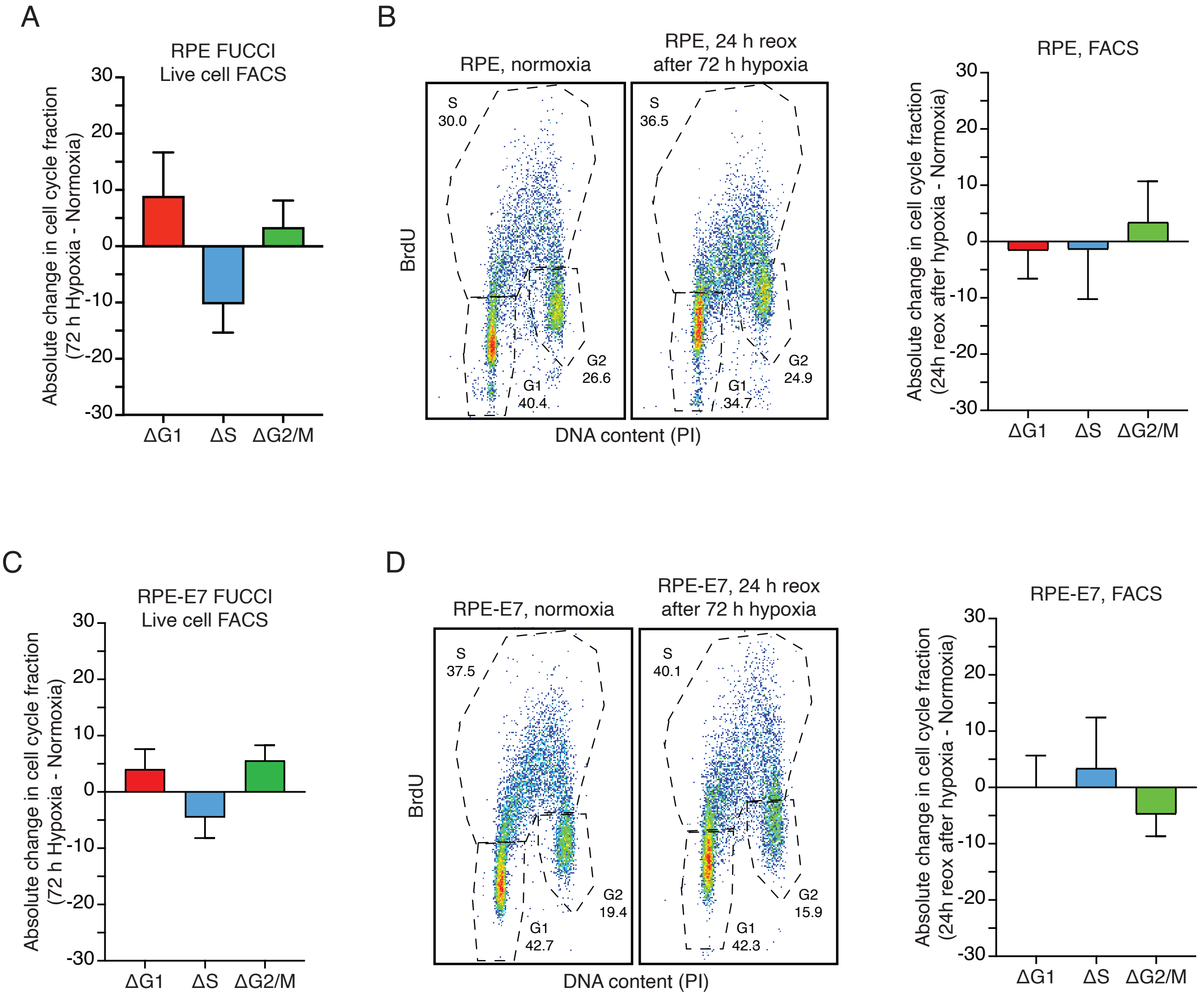

Supplementary Figure 2 - Radiation Sensitivity of proliferating Normoxic and Hypoxic early-G1 cells

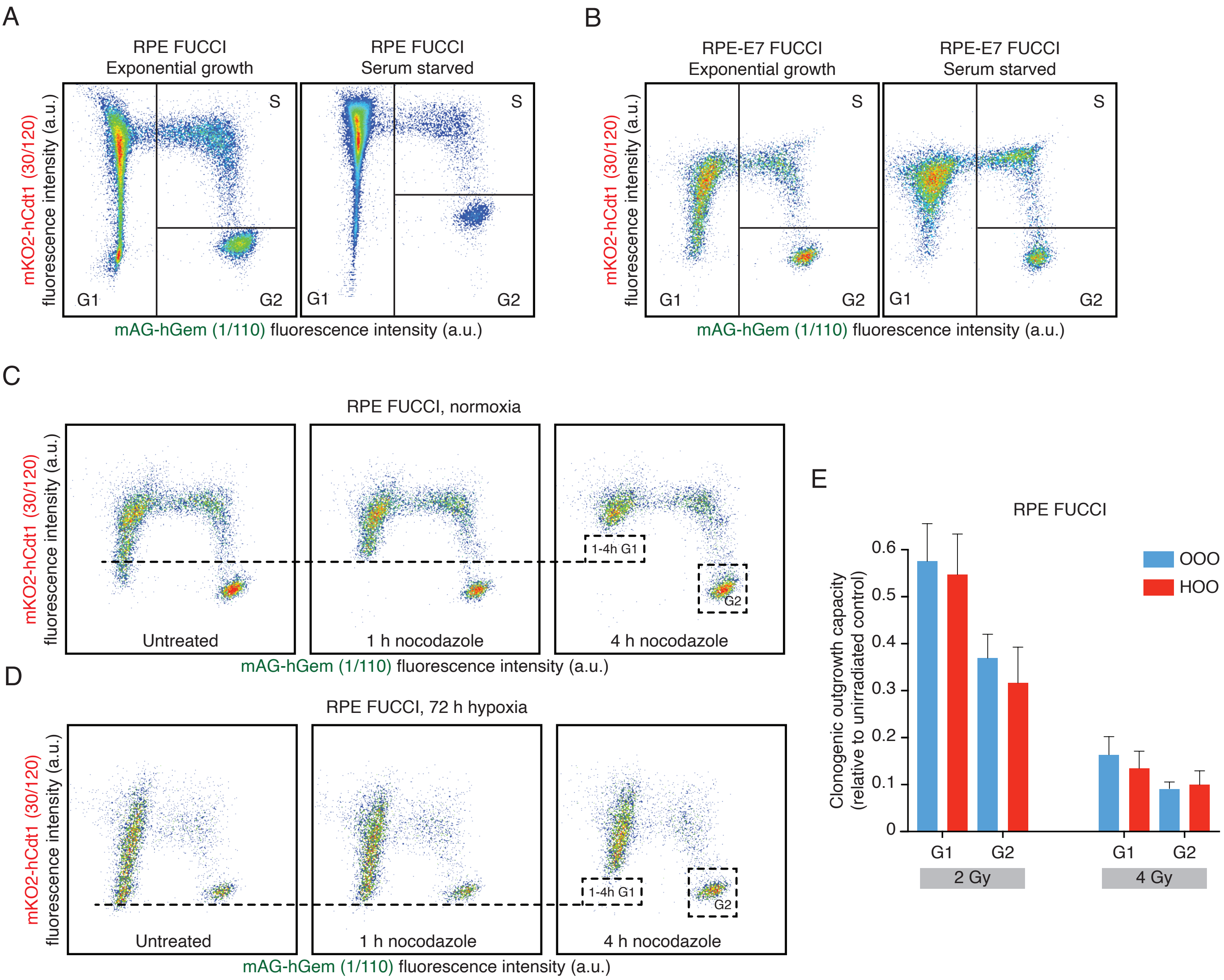

Supplementary Figure 3 - Hypoxia-induced G1 accumulation in tumor cell lines with abberant G1/S transition

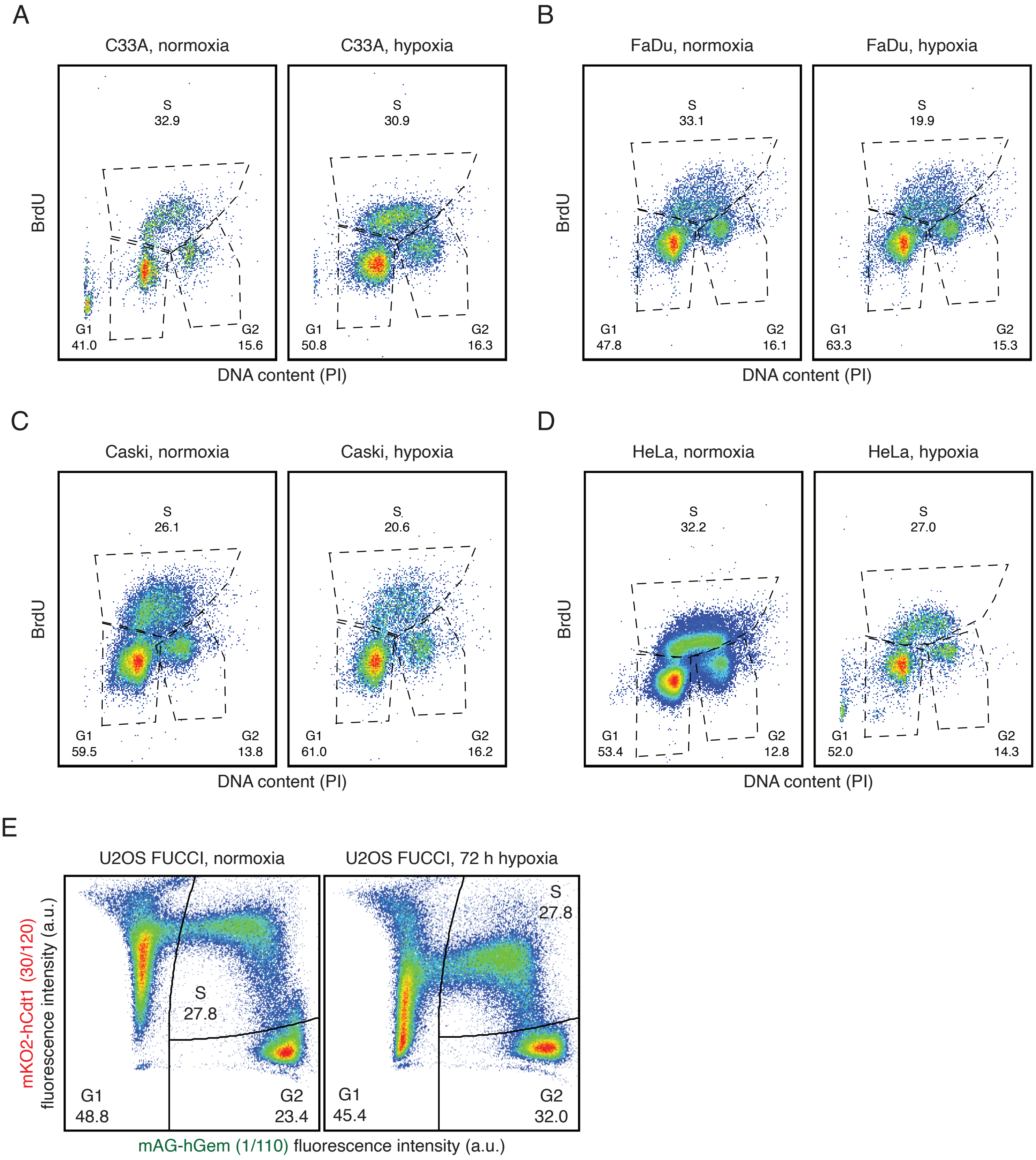

Supplementary Figure 4 - Development of automated macro to assess proliferation and hypoxia pattern in multicellular spheroids.

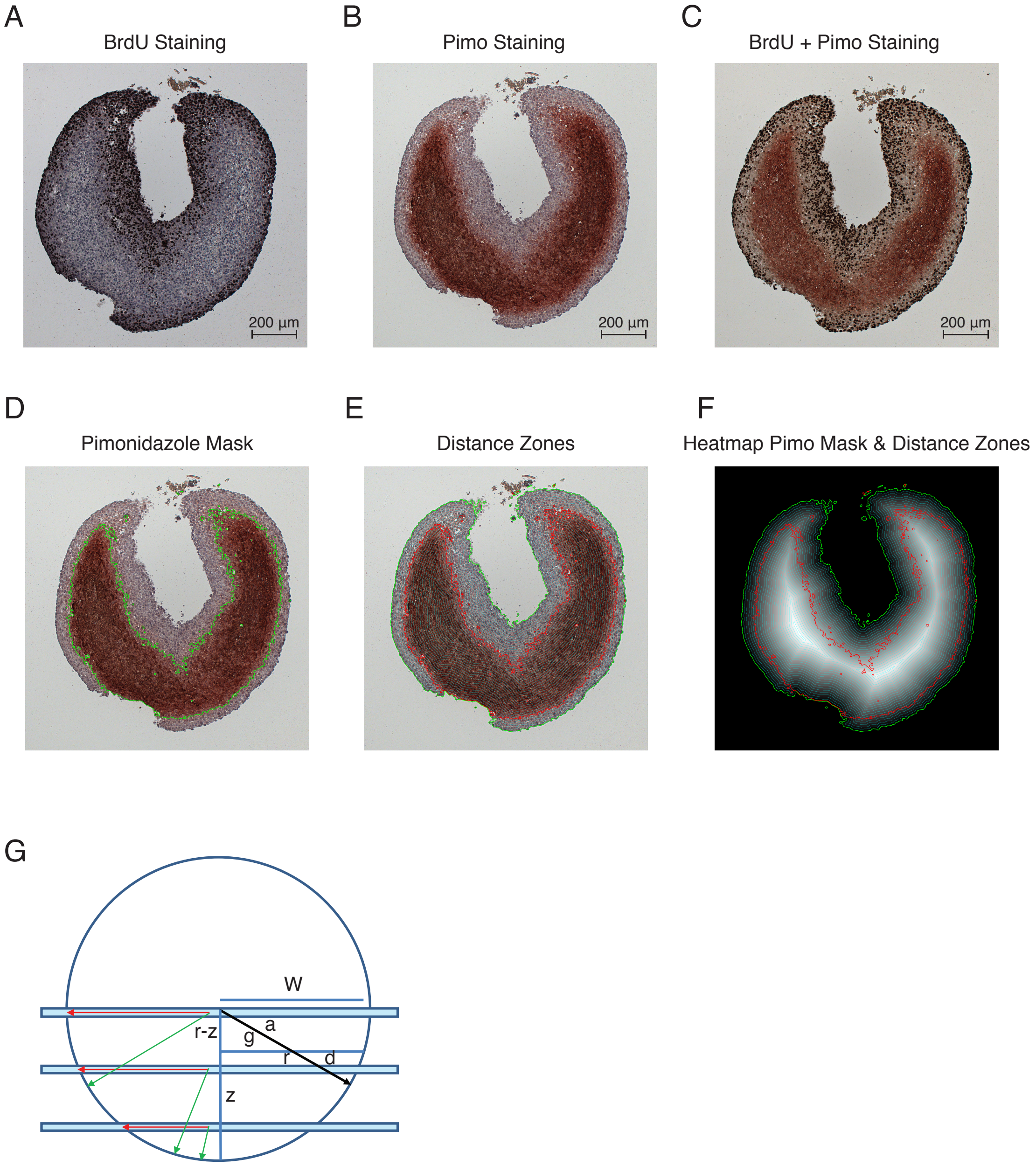

Supplement: Supplementary file 1 [file cells-10-00610-s001.zip › supplementary figures S1-S4.pdf]
